# MiRNA-218 Is Frequently Downregulated in Malignant Breast Tumors: A Footprint of Epstein-Barr Virus Infection

**DOI:** 10.30699/IJP.20201.521107.2550

**Published:** 2021-07-06

**Authors:** Javad Charostad, Mohsen Nakhaei, Azarakhsh Azaran, Gholam Abbas Kaydani, Akram Astani, Azim Motamedfar, Manoochehr Makvandi

**Affiliations:** 1 *Cancer Research Center, Ahvaz Jundishapur University of Medical Sciences, Ahvaz, Iran *; 2 *Department of Virology, School of Medicine, Ahvaz Jundishapur University of Medical Sciences, Ahvaz, Iran*; 3 *Department of Laboratory Sciences, School of Allied Health Sciences, Ahvaz Jundishapur University of Medical sciences Ahvaz, Iran *; 4 *Department of Microbiology, Shahid Sadoghi University of Medical Sciences, Yazd, Iran*; 5 *Department of Nuclear Medicine, School of Medicine, Golestan Hospital, Ahvaz Jundishapur University of Medical sciences, Ahvaz, Iran*

**Keywords:** Breast Neoplasm, Epstein Barr Virus, Human microRNA218

## Abstract

**Background & Objective:**

The role of Epstein-Barr Virus in development of breast cancer is frequently studied. In this regard, miRNAs are among the contributing elements in the molecular pathophysiology of EBV-related diseases. In addition, a growing number of host miRNAs are believed to be implicated in pathogenesis of breast cancer. MiR-218 is a tumor suppressive miRNA that is subjected to dysregulation in various EBV-associated cancers. We aimed to investigate the frequency of EBV and its relationship with expression status of tumor suppressive miR-218 in breast cancer and adjacent normal tissue.

**Methods:**

A total number of 51 fresh malignant breast cancer tissues (cases) and their adjacent normal tissues (controls) were collected. Nested-PCR and RT-qPCR were set to identify EBV frequency and miR-218 expression in cases and controls, respectively.

**Results:**

Out of all samples, 6.8% (7/102) comprising 11.6% (6/51) in malignant tissues and 1.9% (1/51) in normal control tissues were positive for EBV (*P*<0.05). Quantitative data showed that miR-218 was significantly downregulated in malignant tissues compared to control tissues (*P*<0.0001). In addition, reduced expression of miR-218 was associated with adverse clinical outcomes, metastasis, and higher grades of malignancy. Given the presence of EBV, lower expression of miR-218 was observed in breast cancer group in comparison with normal group (*P*<0.05).

**Conclusion:**

Our results raise the possibility of the relation between EBV infection and miR-218 downregulation in breast cancer and propose further investigations in this regard.

## Introduction

Breast cancer is the most prevalent neoplasm in females and the second leading cause of malignancy-related deaths worldwide (1). Increasing incidence and mortality rate of this disease in recent years has turned breast cancer into a major issue for our century (2). Etiologically, breast cancer is considered as a multifactorial phenomenon with multiple internal and external risk factors (3). Among the external risk factors, viral agents such as EBV have been widely implicated in this process (4). In the last three decades, growing interest has focused on the role of viral agents in pathogenesis of breast cancer. 

Epstein Barr Virus (EBV) is a ubiquitous member of the herpesvirus family which is estimated to infect approximately 95% of populations worldwide (5). EBV accounts for approximately 1% of global malignancies with 140 000 related annual deaths (6). EBV infection is strongly involved in a number of human malignancies with epithelial and hematologic origins (7). Epithelial cancers including breast cancer, nasopharyngeal cancer and gastric cancer comprise 80% of all EBV-associated malignancies (8). EBV shows high tropism into these types of the cell, resulting in transformation, permanent proliferation and consequently epithelia cell-derived cancers (9). EBV genome and its products have been frequently detected in cancerous tissues rather than normal breast tissues (10). Oncogenic properties of EBV, which lead to epithelial malignancies are attributed to these products (11).

Numerous studies from various geographical reg-ions such as Asia, Europe and Africa have described the correlation between EBV and breast cancer (9). Several lines of evidence have been indicative of detec-tion of EBV in different subtypes of breast tumors (12). 

The ability of EBV in affecting a series of host cancer-related elements may justify the viral etiology for developing malignancies (7). MicroRNAs (mi-RNAs) are one of the most important molecules among these cancer-related elements.

miRNAs are a group of small single-stranded, non-coding RNAs with 19–23 nucleotides in length (13). miRNAs pervasively orchestrate most physiological and pathological events through their dynamic interplay with mRNA 3′-untranslated region (3′-UTR) (14). A broad variety of miRNAs have been recognized to be engaged in hallmarks of breast carcinogenesis (15). Besides, miRNAs are among the contributing elements in the molecular pathology of EBV-related diseases (16). EBV exploits cellular miRNAs in favor of its pathobiology in infected cells (17). Based on their role in tumorigenesis, miRNAs are characterized into two main classes including tumor-promoting and tumor suppressor miRNAs (18). Mir-218 is a specific intronic miRNA in vertebrates with tumor suppressor functions (19). Mounting evidence reveals that miR-218 is commonly downregulated in different tumors such as prostate, cervical, gastric cancers, nasoph-aryngeal carcinoma, and bladder, glioblastoma, and breast tumors (20). MiR-218 takes part in malignancy-associated characteristics, including apoptosis, differentiation, proliferation, tumor growth and metastasis (20). It is worth noting that miRNA-expression analysis pointed out that miR-218 was underexpressed in specific virus-related cancers (21). 

Studies conducted on clinical samples and ‘in vitro’, reflect significant alterations in expression status of miR-218 in various EBV-associated cancers including nasopharyngeal carcinoma, lymphomas, and gastric cancer (22-24). Existing data have demonstra-ted that downregulation of miR-218 is connected to EB-V infection and adverse malignancy outcomes including invasion, and metastasis. However, the expression status of this miRNA with respect to EBV infection in the context of breast cancer remains unknown.

To our knowledge, this is the first report from Iran that assesses the molecular prevalence of EBV in fresh breast cancer and adjacent normal tissues. Addition-ally, we evaluate the probable correlation between presence of EBV and expression status of miR-218 in breast cancer patients.

## Material and Methods

Study Population

A total of 102 breast tissue biopsies including 51 malignant tissues and their corresponding normal tissues (n=51) were collected from patients who referred to hospitals affiliated to Ahvaz Jundishapur University of Medical Sciences from April 2020 to November 2020.

Specimens were selected based on the pathology reports and were assessed by an expert pathologist. Inclusion criteria were defined as newly diagnosed women with confirmed histopathological evidence of malignancy. The exclusion criteria were defined as the patients with neo-adjuvant treatments, history of cancer, and subjects with other severe organ diseases. All volunteers completed a questionnaire comprising information about lifestyle details, socio-demogra-phics, and medical records. All samples were snap frozen in liquid nitrogen immediately after biopsy pro-cess and stored at −80°C until further analysis. This study has been approved by the Ethics Committee of Ahvaz Jundishapur University of Medical Sciences, Ahvaz, Iran under ethic number IR.AJUMS-.REC.1399.092 and every participant signed a written consent.

DNA Extraction and Qualification

**Fig. 1:**
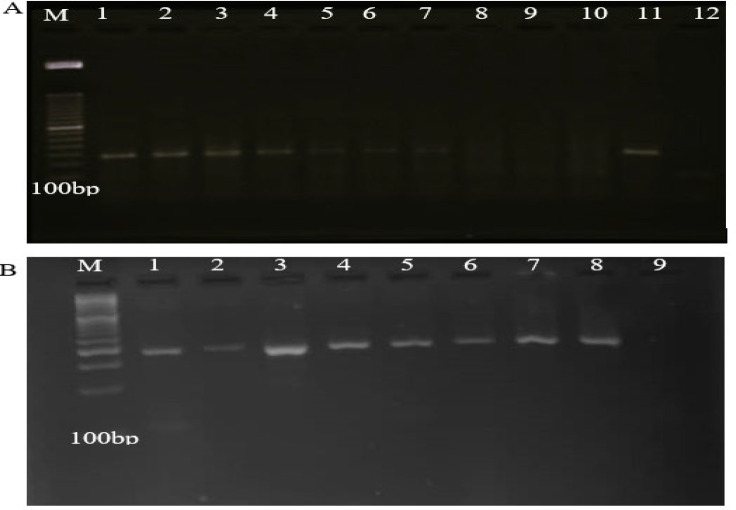
Agarose gel electrophoresis of PCR products. A: amplification of fragment of β-globin: M: 100 bp DNA marker, lanes 1-7: positive samples, lanes 8-10: negative samples, lane 11; positive control, lane 12; Negative control. B: amplification of fragment of EBV EBNA-1 gene: M: 100 bp DNA marker, lanes 1-7: positive samples, lane 8: positive control, lane 9: negative control

DNA from malignant and adjacent normal tissue were extracted using QIAamp DNA Mini Kit (Cat No./ID: 51304) according to the manufacturer’s instructions. Human β-globin amplification (110 bp) was done using the primers PCO3 (5′-ACACAACTGTGTTCACTAGC-3′) and PCO4 (5′-CAACTTCATCCACGTTCACC-3′) to assess the quality of the extracted DNA. β-globin PCR reaction in a final volume of 25 mL contained 1X PCR buffer, 1.5 mM MgCl2, 100 μM dNTPs, 10 pmol of each primer, 1 unit of Taq DNA polymerase, and 500 ng of isolated DNA sample. PCR program was as follow: initial denaturation at 95°C for 10 min, 35 cycles of denaturation at 95°C for 1 min, annealing at 55°C for 1 min, extension at 72°C for 1 min, and final extension at 72°C for 8 min. A negative and a positive sample were used as controls in each PCR test. Subsequently, all PCR products underwent electrophoresis in 2% agarose gel and results were assessed based on the presence of fragments in the expected size (110 bp) (Figure 1A). All specimens which were positive for β-globin gene underwent the following assessments. 

In the present study, strict precautions were taken to avoid contamination in DNA extraction and PCR procedure including utilization of two separate class II laminar flow hoods in physically separate stations that were equipped with UV light, disposable DNase/-RNase free filter tips, and rigid DNA decontamination procedure. All pre-amplification and post-PCR steps were carried out in different rooms and negative and positive control were included in each PCR test.

Detection of EBV Genome

Nested-PCR was applied to identify fragments in conserved regions of EBV EBNA-1 gene using sense and antisense primers including 5′-GTAGAAGGCC-ATTTTTCCAC-3′ and 5′-CTCCATCGTCAAAGCT-GCA-3′ in the first and 5′-AGATGACCCAGGAGA-AGGCCCAAGC-3′and 5′-CAAAGGGGAGACG-ACTCAATGGTGT -3′ in the second PCR. Each PCR mix reactions was performed in a total volume of 25 μL containing 500 ng purified/PCR product DNA, 1X PCR buffer, 1.5 mM MgCl2, 200 μM of each dNTPs, 100 pmol of each primer (the first and the second PCR) and 1 unit of Taq DNA polymerase (Amplicon, Denmark). The Nested-PCR thermal cycling conditions were carried out as follows: 5 min at 94°C, 35 cycles of 30 s at 94°C, 45 s at 54°C and 45 s at 72°C with a final extension of 7 min for amplification followed by checking for the presence of a 609 bp product in the first PCR and 5 min at 94°C, 35 cycles of 30 s at 94°C, 45 s at 63°C and 45 s at 72°C with a final extension of 7 min for amplification followed by checking for the presence of a 308 bp product in the second PCR. 2% agarose gel was prepared and loaded by PCR products along with positive and negative controls and then, visualized under UV light. The DNA from an EBV positive mononucleosis patient’s serum was used as EBV positive control (Figure 1B). EBV PCR products were purified and sequenced using an automated sequencer ABI PRISM 3100 (Applied Biosystems, Foster city, CA).

RNA Extraction and Reverse Transcription Quantitative Polymerase Chain Reaction (RT-qPCR)

RNA extraction of all malignant and normal breast tissues was accomplished by RNA Minipreps kit (Bio Basic, Inc.) according to the Kit’s protocols. The quality of extracted RNA was measured by evaluating the preservation of 28S rRNA and 18S rRNA species on agarose gel and also NanoDrop spectrometer (Thermo Fisher Scientific, USA) based on 260/280 absorbance ratio. BON-Stem miR cDNA synthesis (Cat No: BN-0011.17.2, Iran) was used to generate cDNA from 1 µg isolated RNA template according to manufacturer’s instructions.

RT-qPCR was carried out to assess the expression of miR-218 with the BonMiR RT-qPCR (Cat No: BON209002, Iran) using StepOnePlus™ Real-Time PCR Systems (Applied Biosystems, Foster City-, USA). RT-qPCR reaction system in total volume 12 contained 2 μL of RT product, 0.5 mM of forward and reverse primer and 2× miRNA BonMiR RT-qPCR master mix. The Real-time PCR program for miR-218 was set as 95°C for 5 min followed by 35 cycles of 95°C for 5 s, 59°C for 30 s, and 72°C for 25 s. SNORD47 acted as the internal control to normalize the expressions. *Escherichia coli* miR-39 and no template control (NTC) were respectively used as positive and negative controls in each RT-PCR run. To calculate the relative quantification of miR-218 gene expression, 2^–ΔΔCt ^formula was applied and all reactions were repeated at least two times.

Statistical Analysis

Results were analyzed in GraphPad Prism version 6 (La Jolla, CA, USA) and IBM SPSS version 21.0 (SPSS, Chicago, IL, USA), then compared employing chi-square test. Mann-Whitney U test was run to compare quantitative data. Kruskal-Wallis test was used to assess gene expressions between the groups. The P-value<0.05 (*), P-value <0.01 (**), P-value <0.001(***) and P-value <0.0001(****) were defined as statistically significant.

## Results

**Socio-demographic Status and Clinicopatho-logical Characteristics**

**Fig. 2:**
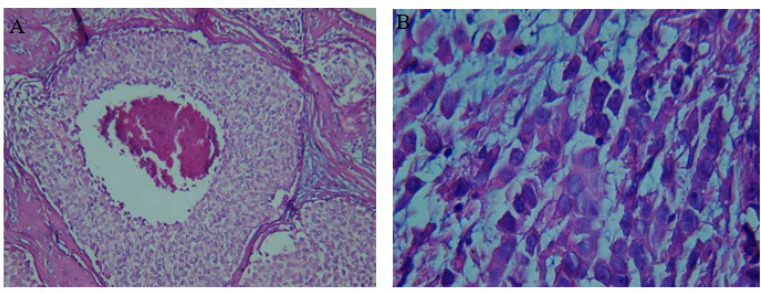
Histological sections of hematoxylin-eosin-stained breast tumor tissue. A: invasive ductal carcinoma with tumor grade III and tumor percentage 50%. B: invasive lobular carcinoma with tumor grade I and tumor percentage 50%

In this study, 102 breast tissue samples were included comprising malignant (cases, n=51) and adjacent non-malignant tissue samples (controls, n=51) obtained from 51 patients. The mean age of volunteers was 50.2±1.4 with a range of 28-68 years. Table 1 presents the patients’ lifestyle parameters, socio-demographic status, and clinical records. In breast cancer patients, 40 had invasive ductal carcinomas (IDC) (78.4%), and 11 had invasive lobular carcinomas (ILC) (21.6%) (Figure 2). Patients’ pathological characteristics are summarized in Table 2.

Detection of EBV

Screening of EBV genome using nested-PCR showed that seven cases (6.8%) out of 102 were positive. Frequency of EBV infection in malignant tissue (n=6, 11.76%) was significantly higher than normal tissue (n=1, 1.9%) (*P*<0.05). There was no significant difference in the frequency of EBV regarding demographic status and clinicopathological data (Table 3).

The Status of miR-218 Gene Expression

**Fig. 3:**
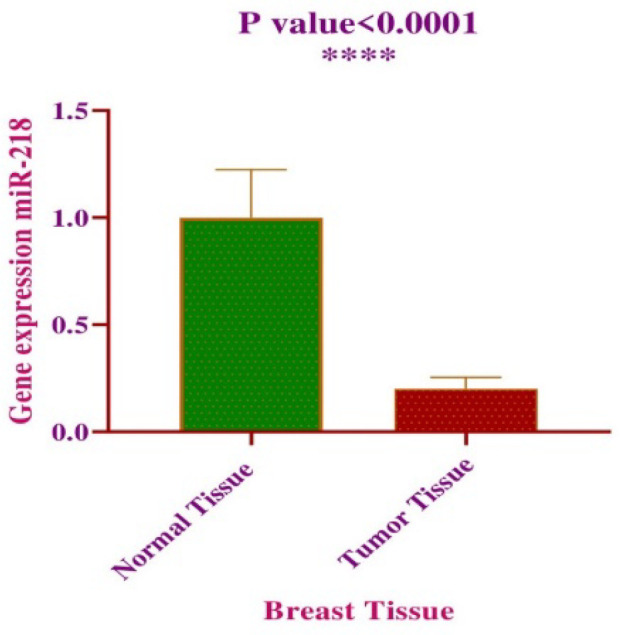
Mean expression level of miR-218 in tumoral and normal tissue

The expression of miR-218 in malignant and adjacent non-malignant tissues was examined by RT-qPCR. Quantitative results of miR-218 relative expression revealed that miR-218 is strongly reduced in malignant tissues compared to cancerous tissues (Figure 3) (*P*<0.0001). In addition, in cases with high histological grades (II/III) and lymphoid metastasis, reduced expression level of miR-218 was observed in comparison with the cases with low histological grades (I) and without lymphoid metastasis (Table 3).

**Fig. 4:**
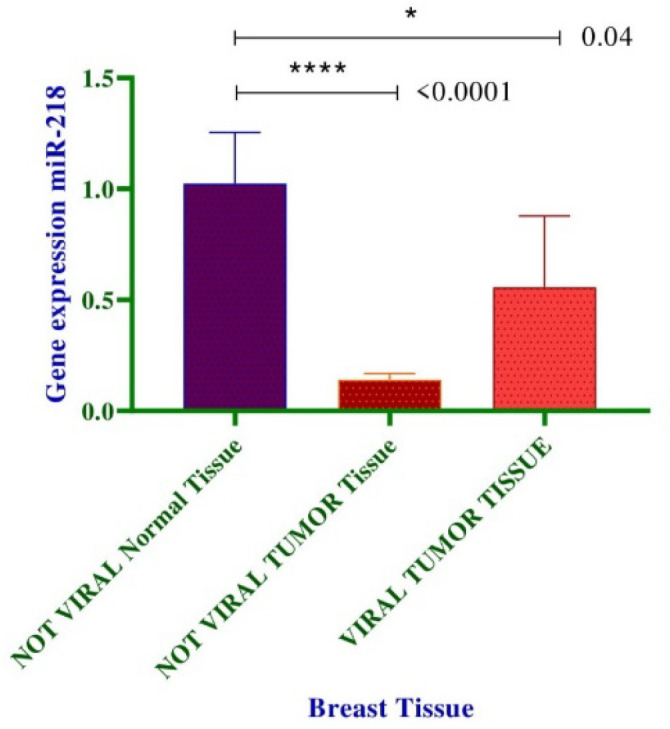
Comparison of miR-218 expression within tumoral and normal tissues in reference to EBV presence.^ *^P<0.05; ^**^P<0.01; ^***^P<0.001; ^****^P< 0.0001

Analysis of miR-218 expression according to presence of EBV infection displayed a direct correlation between viral positivity and reduced expression level of miR-218 (Figure 4, *P*<0.05).

**Table 1:** Socio-demographic parameters and clinical records of all subjects involved in the study

%	Number	Parameter	
**43.1**	22	≤ 45 > 45	**Age**
**56.9**	29		
**51**	26	Primary school High school graduate	**Education**
**35.3**	18		
**13.7**	7		
**74.5**	38	Married Divorced Single	**Marital Status**
**7.8**	4		
**17.6**	9		
**66.7**	34	Yes No	**Children**
**33.3**	17		
**67.6**	23	1-3 > 3	**No. Children**
**32.4**	11		
**72.5**	37	Urban Rural	**Place of residence**
**27.5**	14		
**31.4**	16	Employed Unemployed	**Occupational status**
**68.8**	35		
**27.5**	14	Yes No	**Family history of cancer**
**72.5**	37		
**15.7**	8	Yes No Unknown	History of bacterial STDs*
**76.5**	39		
**7.8**	4		
**5.9**	3	Yes No Unknown	History of viral STDs*
**86.3**	44		
**7.8**	4		
**45.1**	23	Yes No Unknown	**Pap smear**
**45.1**	23		
**9.8**	5		
**100**	15	Normal Abnormal	**Pap smear**
**0**	0		
**5.9**	3	Yes No Unknown	HRSB*
**86.3**	44		
**7.8**	4		
**54.9**	28	Pre Post Unknown	**Menopause**
**37.3**	19		
**7.8**	4		
**88.2**	45	Non- Smoking Ex- Smoking Current	**Smoking**
**7.8**	4		
**3.9**	2		
**5.9**	3	Yes No	**Alcohol consumption**
**94.1**	48		
**58.8**	30	Low Moderate High	**Physical activity**
**35.3**	18		
**5.9**	3		
**43.1**	22	≤ 162 > 162	**Height**
**56.9**	29		
**41.2**	21	≤ 64 > 64	**Weight**
**58.8**	30		
**72.5**	37	20-25 25-30 > 30	**BMI**
**21.6**	11		
**5.9**	3		

Abbreviations: STDs; sexually transmitted diseases, HRSBs; high risk sexual behaviors

**Table 2:** Patients’ pathological-related parameters

%	Number	Parameter	
**78.4**	40	IDC ILC	**Tumor Type**
**21.6**	11		
**31.8**	16	Low (I)High (II/III)	**Histological** **Grade**
**68.2**	35		
**62.7**	32	Early (I/II) Advanced (III/IV)	**Clinical Stage**
**37.3**	19		
**25.5**	13	Yes No	**Lymphoid Metastasis**
**74.5**	38		
**64.7**	33	**+** **_**	**ER**
**35.3**	18		
**43.1**	22	**+** **_**	**PR**
**56.9**	29		
**54.9**	28	**+** **_**	**HER-2**
**45.1**	23		

**Table 3:** EBV positivity and miR-218 expression in breast cancer patients with respect to the demographic and pathological data. Results are expressed as frequencies and mean± SEM

P-value	Mir-218 Expression	P-value	EBV	Parameter	
**0.711**	0.20±0.04	0.718	3	≤ 45 > 45	**Age**
	0.24±0.08		3		
**0.325**	0.22±0.04	0.756	5	IDC ILC	**Tumor Type**
	0.26±0.15		1		
0.041*	0.34±0.11	O.912	2	Low (I) High (II/III)	**Histological** **Grade**
	0.17±0.05		4		
**0.853**	0.24±0.06	0.492	3	Early (I/II) Advanced (III/IV)	**Clinical Stage**
	0.21±0.08		3		
0.048*	0.12±0.05	0.639	2	Yes No	**Lymphoid Metastasis**
	0.26±0.06		4		
**0.515**	0.26±0.07	0.915	4	+ _	**ER**
	0.15±0.04		2		
**0.206**	0.19±0.07	0.606	2	+ _	**PR**
	0.25±0.06		4		
**0.363**	0.24±0.06	0.258	2	+ _	**HER-2**
	0.21±0.07		4		
**0.285**	0.26±0.07	0.173	1	Primary school High school graduate	**Education**
	0.2±0.09		4		
	0.18±0.09		1		
**0.364**	0.25±0.06	0.738	5	Married Divorced Single	**Marital Status**
	0.15±0.07		0		
	0.16±0.07		1		
**0.165**	0.28±0.07	0.663	4	Yes No	**Children**
	0.11±0.02		2		
**0.971**	0.2±0.04	0.422	2	1-3 > 3	**No. Children**
	0.4±0.2		2		
**0.635**	0.19±0.05	0.188	3	Urban Rural	**Place of residence**
	0.3±0.12		3		
**0.106**	0.19±0.1	0.06	4	Employed Unemployed	**Occupational status**
	0.24±0.05		2		
**0.874**	0.23±0.12	0.529	1	Yes No	**Family history of cancer**
	0.22±0.05		5		
**0.89**	0.37±0.21	0.410	0	Yes No Unknown	**History of STD**
	0.2±0.05		5		
	0.11±0.04		1		
**0.931**	0.25±0.2	0.589	0	Yes No Unknown	**History of STD**
	0.23±0.05		5		
	0.11±0.04		1		
**0.320**	0.31±0.1	0.751	3	Yes No Unknown	**Pap smear**
	0.17±0.03		2		
	0.06±0.02		1		
**NA**	0.34±0.05	NA	3	Normal Abnormal	**Pap smear**
	-		0		
**0.931**	0.25±0.2	0.582	0	Yes No Unknown	**HRSB**
	0.23±0.05		5		
	0.11±0.04		1		
**0.606**	0.18±0.03	0.445	4	Pre Post Unknown	**Menopause**
	0.32±0.12		1		
	0.11±0.04		1		
**0.396**	0.24±0.05	0.636	6	Non- Smoking Ex- Smoking Current	**Smoking**
	0.05±0.01		0		
	0.2±0.17		0		
**0.055**	0.4±0.13	0.514	0	Yes No	**Alcohol consumption**
	0.21±0.05		6		
**0.328**	0.26±0.07	0.636	3	Low Moderate High	**Physical activity**
	0.17±0.05		3		
	0.15±0.11		0		
**0.387**	0.21±0.08	0.215	4	≤ 162 > 162	**Height**
	0.24±0.06		2		
**0.924**	0.27±0.1	0.177	4	≤ 64 > 64	**Weight**
	0.19±0.03		2		
**0.075**	0.25±0.06	0.746	5	20-25 25-30 > 30	**BMI**
	0.12±0.05		1		
	0.31±0.02		0		

## Discussion

Breast cancer is the most frequently diagnosed malignancy in female populations. According to estimates by WHO, only in United States in 2020, 1,806,590 new breast cancer cases and 606,520 related deaths are projected to occur (25). EBV is a biological carcinogen, which plays a crucial role in pathogenesis of breast cancer (3). EBV is able to interact with miRNAs in the course of oncogenesis (26). Likewise, a growing number of host miRNAs are believed to be implicated in the context of breast carcinogenesis. Owing to diverse regulatory activities like tumor suppressive functions, miRNAs are considered to be key players with influential effects on development of breast cancer (15).

Since Labrecque *et al.* in 1995 (27) provided evidence reporting the presence of EBV infection in breast tumors, the potential involvement of EBV in the pathogenesis of breast cancer has been extensively investigated. The association between EBV infection and breast carcinogenesis have been reported by multiple observations such as i) EBV persistency in breast tissue, ii) the capacity of EBV genome to stimulate growth of human breast cells, iii) origination of some EBV-related lymphomas from breast tissues, iv) identification of EBV in benign breast tumors in immunocompromised women, etc. (25).

Previous studies have indicated that the prevalence of EBV in breast cancer ranges from 0% to 78%, worldwide (4). These discrepancies may result from sensitivity of detection method, using fresh or paraffin-embedded specimens and differences in geographic regions. In breast cancer, population-based studies have attributed the highest and the lowest frequency of EBV in European and North American population, respectively (35.68% vs 2.86%) (4).

In the current study, we used a sensitive method serving nested-PCR for the conserved region of EBV EBNA-1 gene. We also collected fresh frozen forms of breast tissue comprising malignant tissues and adjacent non-malignant tissues. Overall, 6.8% (7/102) of samples comprised 11.76% (6/51) in malignant tissues and 1.9% (1/51) in adjacent non-malignant tissues found to be positive for presence of EBV genome, which was statistically significant (*P*<0.05). Similar to our results, several case-control reports from Iran and other parts of the world have demonstrated higher prevalence of EBV in malignant tumors as compared with control groups. In several studies, the prevalence rate of EBV in malignant breast tissues and non-malignant control groups were as follows: 9.3% and 0% in a study by Aghdam *et al.* (Iran, 2017), 8% and 0% in a study by Malekpour *et al.* (Iran, 2015), 5.1% and 0% in a study by Saeedi *et al*. (Iran, 2018), 10% and 0% in a study by Antonsson *et al.* (Australia, 2012), 13.6% and 4.5% in a study by Ladera *et al.* (Venezuela, 2017), respectively (28-32). It is noteworthy that despite other investigations, our study used adjacent non-malignant tissues to improve the accuracy of viral causality in the development of malignancy. Additionally, we collected fresh forms of tissue, which minimized the adverse variations after fixations in formalin-fixed specimens. 

Our RT-qPCR data revealed that the expression of miR-218 is considerably decreased in malignant tissues in comparison with non-malignant tissues (Figure 3) (*P*<0.0001). Interestingly, patients with clinical char-acteristics and high histological grade (II/III) and lymphoid metastasis showed reduced expression level of miR-218 compared to low histological grade (I) and without lymphoid metastasis (Table 3). In accordance with these findings, a study conducted by Liu *et al.* on 49 patients with breast cancer in 2016 elucidated that miR-218 expression was significantly reduced in breast cancer tissues compared with matching adjacent normal tissues (33). Moreover, the authors mentioned “in vitro” experiments highlighting that overexpression of miR-218 diminished proliferation and migration of cancerous cells, representing tumor suppressive func-tion of miR-218. In accordance with our clinicopatho-logical findings, several lines of evidence reported aggressive forms of breast cancer to be related to lower expression of miR-218. In 2017, Ahmadinejad *et al.* reported the relationship between miR-218 downre-gulation and poor prognosis of patients with breast cancer (34). They examined 33 matched breast tumors and corresponding adjacent normal tissues and report-ed that the patients with lymph node metastases and higher grades of cancer had reduced levels of miR-218 expression. Existing data have clarified remarkably reduced expression level of miR-218 not only in malignant tissues compared to normal tissues, but also in metastatic cancer cells compared to premalignant cells (20).

With respect to EBV infection status, we importantly observed that lower expression of miR-218 was correlated with presence of EBV infection (Figure 4) (*P*<0.05). Multiple studies, as well as ours, have emphasized on alterations of miR-218 expression in various EBV-associated cancers. An in vitro study exploring the expression of miR-218 and the related effects in nasopharyngeal carcinoma (NPC), reported downregulation of miR-218 and its clinically relevant consequences such as cell survival and migration in EBV-associated NPC (22). In EBV-associated lymph-oma cases, microRNA profiling by deep sequencing has exhibited that miR-218 is subjected to deregulation and decreased expression relative to normal tissues (23). In another study by Kim *et al.* the expression profiles of miR-218 in clinical samples and cell lines of various gastric cancer subtypes was explored (24). Observations represent that expression of miR-218 was significantly reduced in EBV-positive subtypes of gastric cancer compared to normal tissues.

In the present study, the presence of viral infection and miR-218 expression were assessed according to socio-demographic and clinically relevant parameters (Table 3). Analysis proposes these parameters may not affect the status of EBV infection and miR-218 expression.

Compelling data reveals an interaction between viral infections and dysregulated miRNA-expression signatures in human disease, and its characterization provides an approach for global miRNA profiling in virus-associated human diseases (21). The researchers propose that miRNA-expression profiling can be served as a beneficial biomarker to prognosticate dis-ease consequences in virus-infected cells, especially in EBV-associated diseases (21, 35). Therefore, this study may be a step into complete another piece of miRNA-expression profiles in EBV-associated diseases such as breast cancer. 

## Conclusion

Our investigation may provide important clues to EBV pathobiology and the epidemiology of EBV-associated cancers in the course of breast cancer development. The current study may also lead current knowledge toward plausible explanations to miR-218 expression status in EBV-associated breast cancers. Additionally, strong correlation between reduced levels of miR-218 and malignant tumor and adverse clinical outcomes sheds a light on future perspectives and can be considered as a useful biomarker for prognosis of breast cancer patients.
